# Interaction of Gamma-Aminobutyric Acid and Ca^2+^ on Phenolic Compounds Bioaccumulation in Soybean Sprouts under NaCl Stress

**DOI:** 10.3390/plants11243503

**Published:** 2022-12-13

**Authors:** Chong Xie, Maomao Sun, Pei Wang, Runqiang Yang

**Affiliations:** Whole Grain Food Engineering Research Center, College of Food Science and Technology, Nanjing Agricultural University, Nanjing 210095, China

**Keywords:** GABA, Ca^2+^, phenolic compounds, soybean sprout, NaCl stress

## Abstract

NaCl stress can enhance the accumulation of phenolic compounds in soybean during germination. In the present study, effects of gamma-aminobutyric acid (GABA) and Ca^2+^ on the biosynthesis of phenolic compounds in soybean sprouts germinated with NaCl stress were investigated. Results showed that addition of Ca^2+^ increased the content of total phenolics, phenolic acids, and isoflavonoids in soybean sprouts by ca. 15%, 7%, and 48%, respectively, through enhancing the activities of three key enzymes involved in the biosynthesis. On the other hand, addition of LaCl_3_, a calcium channel blocker, inhibited the synthesis of phenolic compounds, indicating that Ca^2+^ plays an important role in the synthesis of these compounds in soybean sprouts. Addition of GABA can increase the content of Ca^2+^ in soybean sprouts by ca. 20% and alleviate the inhibition of LaCl_3_ on phenolics biosynthesis in soybean sprouts. Similarly, addition of Ca^2+^ can reverse the inhibition of 3-mercaptopropionate, an inhibitor of endogenous GABA synthesis, on the biosynthesis of phenolic compounds in soybean sprouts under NaCl stress. To conclude, both GABA and Ca^2+^ can enhance the biosynthesis of phenolic compounds in soybean sprouts and there was an interaction between their effects on the promotion of phenolic compounds biosynthesis.

## 1. Introduction

Phenolic compounds are molecules consisting of one or more hydroxyl groups bonded to the aromatic hydrocarbon group, which have various biological activities, including antioxidant, antimicrobial, and anti-inflammatory properties [1]. Developing foods rich in phenolic compounds is becoming increasingly popular all around the world because of their health benefits. Soybean (*Glycine max* L.) is a valuable crop playing a substantial role in both food and feed production because it is rich in protein, lipids, and various functional components [2]. During soybean germination, various nutrients are accumulated, including phenolic compounds. Considering the consumption of soybean sprout products is common in many areas, especially among Asian countries, the enrichment of phenolic compounds in soybean through germination is a promising and cost-effective way to provide these nutrients to consumers.

As the secondary metabolites in plants, phenolic compounds participate in responses to many types of abiotic stress, such as drought, extreme temperatures, anoxia, salinity, and herbicides [3,4]. Salt stress, as an abiotic stress, can result in the accumulation of reactive oxygen species (ROS) content in sprouts, which will induce the accumulation of antioxidants, including phenolic compounds [5]. Therefore, salt stress, such as NaCl treatment, has been widely used to enhance the accumulation of phenolic compounds in edible sprouts, such as barley [6], buckwheat [7], and radish [8]. However, the mechanism of phenolics accumulation during germination in NaCl stress has not been fully elucidated. 

Gamma-aminobutyric acid (GABA) is a ubiquitous four-carbon signal molecule with versatile roles in the metabolism of plants, including the response to abiotic and biotic stress, maintaining carbon/nitrogen balance, and regulating plant development [9]. Our previous works have found that GABA can influence the synthesis of phenolics and antioxidant systems in soybean germinated under NaCl stress [10] and nitric oxide, a signal molecule involved in various responses to stress, can mediate the signaling effect of GABA during phenolic biosynthesis [11]. In plants, calcium is not only a nutrient, but also functions as a second messenger in the metabolism [12]. Upon biotic or abiotic stresses, the concentration of Ca^2+^ in the cytoplasm increases rapidly and interacts with sensor responders or sensor relays to produce signals with the help of some target proteins, such as calmodulin (CaM) [13].

Whether there is an interaction between calcium and GABA in the regulation of phenolic compounds biosynthesis in soybean during germination under salt stress is still unknown. In the present study, calcium (CaCl_2_) and lanthanum (III) chloride (LaCl_3_), a calcium channel blocker, as well as GABA and the inhibitor of GABA biosynthesis, 3-mercaptopropionate (3-MP), were applied to soybean seeds during germination with NaCl stress. Content of endogenous calcium, total phenolic compounds, phenolic acids, and isoflavonoids, as well as activities of key enzymes during biosynthesis were determined to elucidate the effects of calcium and GABA on the synthesis of phenolic compounds.

## 2. Results 

### 2.1. Content of Total Phenolic Compounds in Sprouts

**Figure 1:**
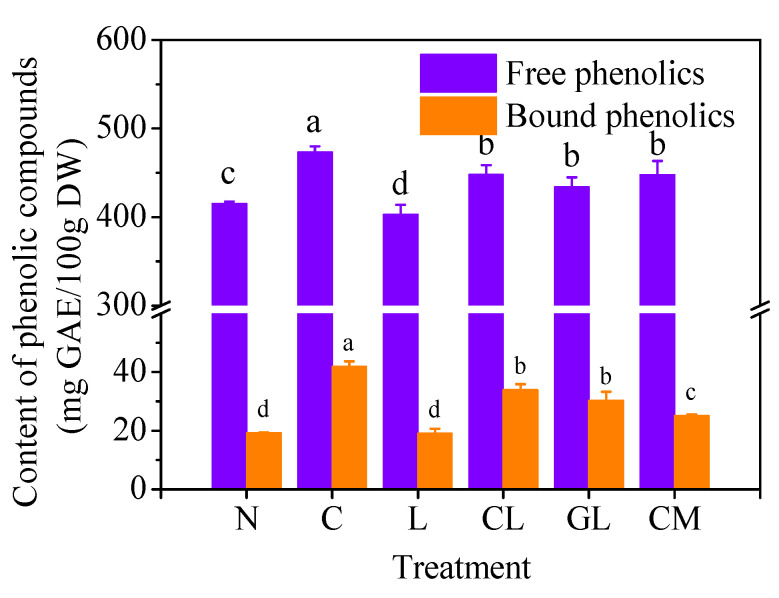
Content of phenolic compounds (mg GAE/100 g DW) in soybean sprouts (n = 3). Treatments were as follows: N: NaCl; C: NaCl + CaCl_2_; L: NaCl + LaCl_3_; CL: NaCl + CaCl_2_ + LaCl_3_; GL: NaCl + GABA + LaCl_3_; CM: NaCl + CaCl_2_ + 3-mercaptopropionate (3-MP). Values bearing different letters in each form are significantly different (*p* < 0.05).

In the present study, the contents of free and bound phenolics in the sprout treated with NaCl (N sprout) were 409 mg GAE/100g DW and 20 mg GAE/100g DW, respectively (Figure 1). Addition of Ca^2+^ (C sprout) significantly (*p* < 0.05) increased the content of both free (ca. 470 mg GAE/100g DW) and bound phenolics (42 mg GAE/100g DW) and addition of LaCl_3_ (L sprout) significantly (*p* < 0.05) decreased the content of free phenolics to 350 mg GAE/100g DW in sprout germinated under NaCl stress. The sprout treated with NaCl, Ca^2+^, and LaCl_3_ had significantly (*p* < 0.05) higher levels of free (450 mg GAE/100g DW) and bound phenolic compounds (32 mg GAE/100g DW) than the N sprout. The sprout treated with NaCl, GABA, and LaCl_3_ (GL sprout) had a similar level of bound and free phenolics with the CL sprout. The sprout treated with NaCl, CaCl_2_, and 3-MP (CM sprout) had a similar level of free phenolics with CL and GL sprouts but a significant lower content of bound phenolics.

### 2.2. Content of Phenolic Acids and Isoflavonoids in Sprout

Six kinds of phenolic acids (*p*-hydroxybenzoic acid, vanillic acid, syringic acid, *p*-coumaric acid, ferulic acid, and sinapic acid) were determined in the present study and the free form of *p*-coumaric acid was the dominant type of phenolic acid among all the sprouts (Table 1). The content of total phenolic acids (free plus bound) in the N sprout was 2340 µg/g DW. The highest content of free and bound forms of phenolic acid were observed in the GL sprout (1992 µg/g DW) and C sprout (677 µg/g DW), respectively, and these two sprouts both had the highest level of total phenolic acids. The L sprout had the lowest level of both free (1570 µg/g DW) and bound phenolic acids (176 µg/g DW).

Eight kinds of isoflavonoids (daidzin, glycitin, genistin, malonydaidzin, malony glycitin, malonygenistin, daidzein, and genistein) were determined as shown in Table 2. Malonyldadzein and malonylgenistein were the major forms in all sprouts, which constituted more than 80% of total isoflavonoids content in sprouts. The content of total isoflavonoids in all sprouts ranged from 6236.06 ± 14.71 µg/g DW (L sprout) to 9515.42 ± 61.83 µg/g DW (C sprout). The content of total isoflavonoids in N sprout was ca. 6447 µg/g DW. The content of total isoflavonoids in the CL sprout (8016 µg/g DW) was significantly (*p* < 0.05) higher than the GL (7687.9 ± 40.3 µg/g DW) and CM sprouts (7281.6 ± 45.8 µg/g DW).

### 2.3. Activities of Key Enzymes Involved in Phenolic Compounds Synthesis

**Figure 2:**
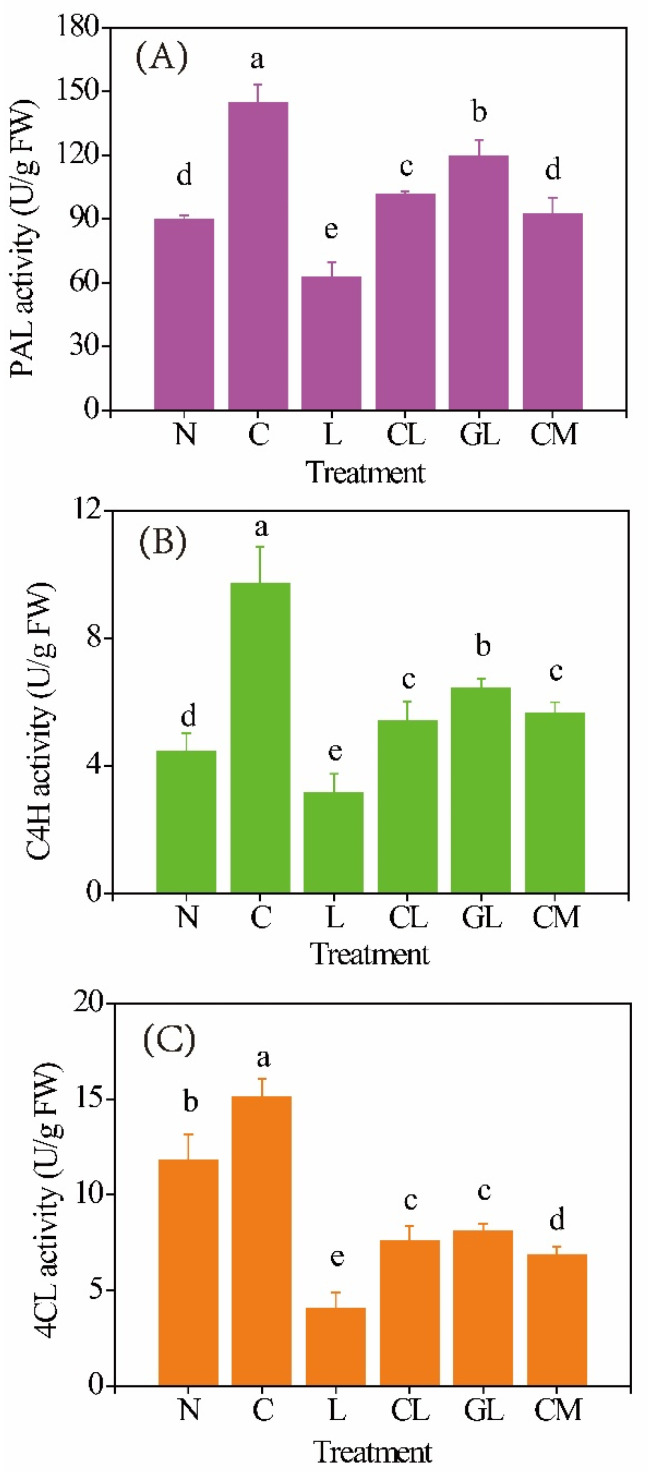
Activities of PAL (**A**), C4H (**B**), and 4CL (**C**) in soybean sprouts (n = 3). N: NaCl; C: NaCl + CaCl_2_; L: NaCl + LaCl_3_; CL: NaCl + CaCl_2_ + LaCl_3_; GL: NaCl + GABA + LaCl_3_; CM: NaCl + CaCl_2_ + 3-mercaptopropionate (3-MP). Values bearing different letters in each line are significantly different (*p* < 0.05).

Activities of three key enzymes, i.e., phenylalanineammonialyas (PAL), cinnamate 4-hydroxylase (C4H), and 4-coumarate-CoA ligase (4CL), involved in phenolics biosynthesis in sprouts are shown in Figure 2. C sprout had the highest activities of PAL (144 U/g FW), C4H (15.1 U/g FW), and 4CL (9.7 U/g FW). L sprout had the lower activities of these enzymes, which were 63 U/g FW (PAL), 3.1 U/g FW (C4H), and 4.1 U/g FW (4CL), respectively. CL and GL sprouts had significantly (*p <* 0.05) higher activities of PAL and C4H but a lower activity of 4CL than N sprout. CM sprout had almost the same level of PAL activity with the N sprout.

### 2.4. Content of Ca^2+^ and CaM

**Figure 3:**
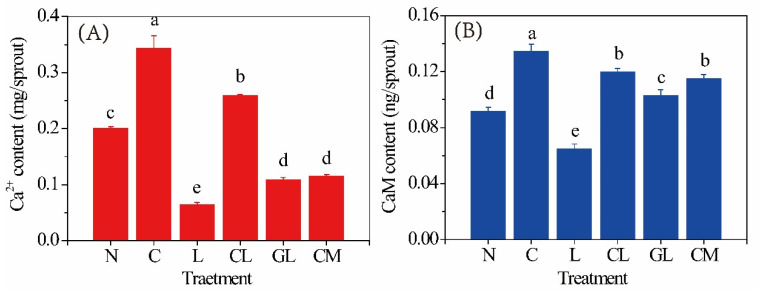
(**A**) Content of Ca^2+^ (mg/sprout) and (**B**) calmodulin (CaM; ng/sprout) in soybean sprouts (n = 3). Sprouts in all treatments had a dry weight at around 0.12 g. Treatments were as follows: N: NaCl; C: NaCl + CaCl_2_; L: NaCl + LaCl_3_; CL: NaCl + CaCl_2_ + LaCl_3_; GL: NaCl + GABA + LaCl_3_; CM: NaCl + CaCl_2_ + 3-mercaptopropionate (3-MP). Values bearing different letters are significantly different (*p* < 0.05).

N sprout had 0.2 mg/sprout of Ca^2+^ (Figure 3A) and 0.09 ng/sprout of CaM (Figure 3B). C sprout had the highest content of Ca^2+^ (0.34 mg/sprout) and CaM (0.13 ng/sprout) among all the sprouts. The lowest content of both Ca^2+^ (0.06 mg/sprout) and CaM (0.06 ng/sprout) were found in L sprout. GL sprout had significantly (*p* < 0.05) higher levels of Ca^2+^ (0.11 mg/sprout) and CaM (0.09 ng/sprout) than L sprout. CM sprout had a significantly (*p* < 0.05) lower level of Ca^2+^ content (0.12 mg/sprout) than C sprout.

## 3. Discussion

Phenolic compounds, including phenolic acids, tannins, lignans, and flavonoids, are secondary metabolites ubiquitously present in plants with many functions [14]. Improving the phenolic compounds in plant tissues markedly increases the antioxidant capacity and machinery which enhances the plant growth even under various environmental stresses [15,16,17,18]. In plants, phenolic compounds are mainly synthesized from phenylpropanoid pathways, which are initiated by the transformation of phenylalanine to *trans*-cinnamic acid by the action of PAL [19]. Then, cinnamic acid will be hydroxylated by the action of C4H to form *p*-coumaric acid, which will form *p*-coumaroyl CoA, the pivotal molecule to form caffeoylquinic acids and its derivatives, by catalyzation of 4CL [20]. Therefore, PAL, C4H, and 4CL are key enzymes that are involved in controlling the biosynthesis of phenolic compounds. 

Ca^2+^ is a versatile signaling molecule in the plant metabolism and is at the core of cellular responses to adverse environmental conditions [21]. Change of Ca^2+^ content in the cytoplasm is the earliest response of cells to abiotic stresses and CaM is the main calcium sensor in plants [21]. In the present study, it was found that the application of exogenous Ca^2+^ enhanced the accumulation of phenolic compounds in soybean sprout during germination with NaCl stress (Figure 1). The promotion of phenolics accumulation can be explained by the increase in PAL, C4H, and 4CL activities (Figure 2). Meanwhile, activities of these three enzymes were significantly (*p* < 0.05) inhibited by the decrease of endogenous Ca^2+^ (Figure 3) caused by addition of LaCl_3_, the inhibitor of influx of calcium into the cell. These results indicated Ca^2+^ functioned as a signal mediator in the synthesis of phenolic compounds by influencing the activities of the key enzymes. 

It is well known that the Ca^2+^/calmodulin system can activate glutamate decarboxylase in the cytosol and concomitantly increase the content of GABA [22]. On the other hand, GABA, as a versatile signaling molecule, can mediate the uptake or accumulation of minerals in plants. For instance, it was reported that application of GABA during germination can reduce the uptake of toxic heavy metals, such as arsenic [23] and cadmium [24]. However, the effect of GABA on the content of endogenous calcium in sprouts germinated under abiotic stress has not been well studied. In the present study, the sprout treated with NaCl, GABA, and LaCl_3_ (GL sprout) had significantly (*p* < 0.05) higher levels of Ca^2+^ (ca. 0.11 mg/sprout) and CaM (ca. 0.09 ng/sprout) than the L sprout, which indicated that addition of GABA can promote the uptake of calcium by alleviating the inhibition of LaCl_3_ on Ca^2+^ absorption. Meanwhile, a significantly (*p* < 0.05) lower level of Ca^2+^ content (ca. 0.12 mg/sprout) was observed in the sprout treated with NaCl, CaCl_2_, and 3-MP (CM sprout) than the C sprout suggesting that inhibition of the synthesis of endogenous GABA can decrease the Ca^2+^ content of soybean sprout. Meanwhile, addition of GABA can reverse the reduction of endogenous Ca^2+^ content and inhibition on PAL, C4H, and 4CL activities caused by LaCl_3_. 

Phenolic acids and isoflavonoids were the main types of phenolic compounds in soybean sprouts. After germination, contents of phenolic acids in the free form were much higher than the bound form in soybean sprouts, which was not in accordance with a study in the barley sprout [25]. A possible explanation for this difference is that phenolic acids in barley are mainly bound to arabinoxylans [26], while in soybean sprouts, phenolic acid was mainly in esterified forms [27], which is easier to be released in its free form during germination. Isoflavonoids are a class of flavonoids that widely exist in soybean seeds with many beneficial properties, such as antioxidant and anticancer properties [28]. Malonyldadzein and malonylgenistein were the dominant forms of isoflavonoids in the soybean sprout and addition of Ca^2+^ enhanced their accumulation, but the promotion on malonyldadzein (by ca. 100%) was much stronger than malonylgenistein (by ca. 9%). Our previous study showed GABA treatment enhanced the accumulation of malonylgenistein by ca. 60% but had no effect on malonyldadzein [10]. However, addition of LaCl_3_ (5 mM) resulted in a significant (*p* < 0.05) decrease of isoflavonoids (Table 2). It was reported that a low level (ca. 0.08 mM) of LaCl_3_ addition can promote the accumulation of total flavonoids by enhancing activities of PAL in soybean sprout treated with UV-B radiation [29]. Interestingly, addition of GABA and LaCl_3_ resulted in a significant (*p* < 0.05) increase of both malonyldadzein and malonylgenistein, which indicated the influence of LaCl_3_ on isoflavonoids accumulation is dose-dependent and GABA treatment can alleviate the inhibition of LaCl_3_ on isoflavonoids biosynthesis. These results suggested both GABA and Ca^2+^ can influence the synthesis of isoflavonoids and there was an interaction between these two signaling molecules.

## 4. Materials and Methods

### 4.1. Materials 

Soybean seeds (Dongsheng No. 1) were harvested in 2019, Heilongjiang province, China and stored at −20 °C before use. All the chemicals were analytical-grade reagents and were purchased from Shoude Biotechnology Co., Ltd. (Nanjing, Jiangsu, China). 

### 4.2. Experimental Design 

After rinsing with distilled water, soybean seeds were treated with a 0.5% (*w*/*v*) sodium hypochlorite solution for 15 min and soaked in distilled water (1:4, *w*/*v*) at 30 °C for 6 h. The soaked seeds were placed evenly on the seedling tray and germinated in the incubator (LB-300-II, Longyue Instrument Equipment Co., Ltd., Shanghai, China) at 30 °C. The experiment was planned following a completely randomized design (CRD), with three replicates for each treatment. The seeds were sprayed with different culture solutions for 1 min per hour as follows: N: NaCl (40 mM); C: NaCl (40 mM) + CaCl_2_ (6 mM); L: NaCl (40 mM) + LaCl_3_ (5 mM); CL: NaCl (40 mM) + CaCl_2_ (6 mM) + LaCl_3_ (5 mM); CM: NaCl (40 mM) + CaCl_2_ (6 mM) + 3-MP (0.2 mM); GL: NaCl (40 mM) + GABA (5 mM) + LaCl_3_ (5 mM). The sprouts were collected after 4 days of germination and all had a dry weight at around 0.12 g.

### 4.3. Determination of Total Phenolic Compounds and Phenolic Acids

Extraction of free and bound phenolic compounds was conducted according to Chen et al. [30] with some modifications. The freeze-dried samples were milled and the powders (ca. 2 g) screened by a 60-mesh sieve were degreased by hexane. After they were dried with nitrogen flow, the residues were mixed with methanol (80%, *v*/*v*) and shaken (200 rpm) at 25 °C for 1 h under N_2_ and in the dark. Then, the mixtures were centrifuged (10,000× *g*, 4 °C) for 15 min and supernatants from three times of centrifuging were combined and dried in a rotary evaporator at 40 °C after filtration, which were redissolved with methanol (50%, *v*/*v*) and filled with N_2_ as the free phenolics extract. The residues from the free phenolics extractions were hydrolyzed with NaOH (2 mol/L) in a shaker (25 °C, 200 rpm) for 4 h under darkness. The hydrolysates were adjusted to an acidic pH (1.5 to 2.0) with HCl (6 M). After extraction by ethyl acetate for 15 min, the mixtures were centrifuged (10,000× *g*, 4 °C) for 5 min. After three times of centrifuging, the collected ethyl acetate layers were combined in a flat-bottomed flask and dried in a rotary evaporator at 40 °C. The dried extracts were redissolved in 10 mL with 50% methanol (*v*/*v*) and filled with N_2_ as the bound phenolics extract.

The Folin phenol method was used for determination of total phenolic content according to our previous study [31]. Folin phenol reagent was mixed with the free or bound phenolics extract and maintained for 5 min after vortex. Then, Na_2_CO_3_ solution (75 g/L) was added to the mixture and incubated (25 °C) in the dark for 2 h. After that, the absorbance (765 nm) was determined with a 50% methanol solution as the control and gallic acid was used to make the standard curve. The contents of total free or bound phenolic compounds were expressed as mg GAE/100 g DW.

The contents of free and bound phenolic acids in the extract were determined by a Shimadzu (LC-20A) high-performance liquid chromatography (HPLC, Phenomenex, Torrance, CA, USA) equipped with a C18 column (110A 5 μm particle size, 4.6 × 150 mm). The mobile phases were water and methanol, which both contained 0.1% acetic acid. The flow rate was set at 0.9 mL/min and the column temperature was 35 °C. The phenolic acid content was measured at a wavelength of 280 nm and calculated based on the standard curve (0–90 μg/mL) of each acid.

### 4.4. Determination of Isoflavonoids Content

The identification and quantification of isoflavonoids were determined as described by Jiao et al. [32]. Briefly, the freeze-dried sprouts were ground and mixed with 80% methanol. After incubation (50 °C) for 12 h, mixtures were centrifuged and the supernatants were filtered (0.45 μm). An HPLC (Agilent 1200, Agilent Technologies Co., Ltd., Shanghai, China) equipped with a Zorbax SB-C18 column (5 μm particle size, 4.6 × 150 mm) and a VWD detector (260 nm) was used for the analysis. The mobile phases were water and methanol, which both contained 0.1% acetic acid. Quantification of isoflavonoids was conducted according to the standard curve (0–65 μg/mL).

### 4.5. Determination of Activities of Key Enzymes for Phenolics Synthesis

Activities of three key enzymes, i.e., PAL, C4H, and 4CL, were determined by the spectrophotometric assays as described in the previous study [32]. For the PAL activity determination, ground fresh samples were homogenized with 80% acetone (1:10 *w*/*v*). After freezing (15 min), mixtures were filtered and the dried pellets were mixed with 0.1 M sodium borate buffer (pH 8.8) containing 5 mM β-mercaptoethanol (β-ME), 2 mM ethylenediaminetetraacetic acid (EDTA), and 4% (*w*/*v*) polyvinyl pyrrolidone (PVP), and maintained at 4 °C for 1 h. Then, the homogenates were centrifuged at 10,000× *g* (30 min, 4 °C) and the supernatants were reacted with the solution containing 30 μM l-phenylalanine and 30 mM sodium borate buffer (pH 8.8) for 90 min at 30 °C with shaking. Production of cinnamate during incubation was measured by the absorbance change at 290 nm. For determination of C4H activity, fresh samples were mixed with 0.1 M phosphate buffer (pH 7.6) containing 2 mM β-ME, 0.5 mM EDTA, and 0.25 M sucrose, and ground at 4 °C. The supernatants of the mixtures after centrifugation (10,000× *g*, 30 min, 4 °C) were reacted with 50 mM trans-cinnamic acid in 0.1 M phosphate buffer at 30 °C for 60 min before determination of absorbance at 340 nm. For determination of 4CL activity, fresh samples were mixed with 50 mM Tris–HCl buffer (pH 8.9) containing 15 mM β-ME, 10 µM leupeptin, 4 mM MgCl_2_, 1 mM benzylsulfonyl fluoride, 5 mM ascorbic acid, 10% glycerol, and 0.15% (*w*/*v*) PVP, and ground at 4 °C. Then, mixtures were centrifuged (10,000× *g*, 20 min, 4 °C) and supernatants were reacted with the solution containing 0.6 mM *p*-cumaric acid, 5 mM adenosine triphosphate and MgCl_2_, and 0.4 mM coenzyme A for 20 min at 40 °C before determination of absorbance at 333 nm. The unit of activity for each enzyme was calculated based on the change of absorbance during enzymatic reaction and defined as an increase of 0.01 in absorbance in the reaction of soybean sprouts (per gram, fresh weight) per min. 

### 4.6. Determination of Ca^2+^ Content and CaM Content 

The content of Ca^2+^ was determined by a Ca^2+^ assay kit, which was purchased from Aoqing biotechnology Co., Ltd., Nanjing, China (cat. number MB-0028-2), according to the protocol. The content of CaM was determined by the CaM ELISA assay kit (Jiancheng Bioengineering Institute, cat. Number: MBE21030) according to the protocol.

### 4.7. Data Processing and Statistical Analysis

The results were presented as means and standard deviations (SD) of three biological replicates. The Duncan’s multiple range test was used for the significance (at the 0.05 level) test by SPSS 19.0 (SPSS Inc., Chicago, IL, USA) at a *p* < 0.05. 

## 5. Conclusions

During germination in NaCl stress, application of exogenous Ca^2+^ to soybean can enhance the accumulation of total phenolic compounds, phenolic acids, and isoflavonoids by elevating activities of key enzymes involved in their synthesis (PAL, C4H, and 4CL). Reduction of endogenous Ca^2+^ resulted in a lower accumulation of these substances but addition of GABA can alleviate the inhibition. Meanwhile, exogenous Ca^2+^ can also alleviate the inhibition of 3-MP on the synthesis of phenolic compounds. This study proved there was an interaction between Ca^2+^ and GABA on the synthesis of phenolic compounds but the molecular mechanism of the interaction should be revealed in future studies.

## Figures and Tables

**Table 1:** Contents of individual and total phenolic acids (µg/g DW) during germination (n = 3) in soybean sprouts.

Phenolic Acid	Treatment	Free Form	Bound Form	Total
*p*-hydroxybenzoic acid	N	28.23 ± 0.45 ^c^	3.41 ± 0.14 ^b^	31.64 ± 0.31 ^d^
	C	45.94 ± 1.24 ^a^	5.09 ± 0.76 ^a^	51.03 ± 0.49 ^a^
	L	26.80 ± 0.15 ^d^	1.93 ± 0.03 ^d^	28.73 ± 0.12 ^e^
	CL	42.04 ± 0.21 ^b^	2.54 ± 0.08 ^c^	44.58 ± 0.23 ^c^
	GL	41.62 ± 0.32 ^b^	3.63 ± 0.22 ^b^	45.32 ± 0.47 ^b^
	CM	26.01 ± 0.52 ^d^	2.44 ± 0.05 ^c^	28.48 ± 0.40 ^e^
Vanillic acid	N	129.08 ± 0.15 ^d^	62.77 ± 2.18 ^c^	191.84 ± 2.03 ^d^
	C	183.22 ± 4.46 ^a^	99.62 ± 3.74 ^a^	282.84 ± 8.21 ^a^
	L	71.84 ± 0.23 ^f^	19.04 ± 0.04 ^e^	90.88 ± 0.26 ^f^
	CL	164.81 ± 0.08 ^c^	55.10 ± 7.18 ^c^	219.91 ± 7.10 ^c^
	GL	174.80 ± 0.32 ^b^	55.91 ± 4.68 ^c^	230.71 ± 5.00 ^b^
	CM	100.89 ± 0.05 ^e^	36.16 ± 0.10 ^d^	137.05 ± 0.05 ^e^
Syringic acid	N	265.03 ± 1.68 ^d^	295.37 ± 5.52 ^a^	560.40 ± 4.84 ^c^
	C	371.51 ± 5.19 ^a^	278.34 ± 19.51 ^b^	649.86 ± 14.31 ^a^
	L	363.01 ± 8.76 ^a^	33.84 ± 0.46 ^e^	396.85 ± 8.30 ^e^
	CL	300.17 ± 0.05 ^c^	299.70 ± 7.12 ^a^	599.86 ± 7.17 ^b^
	GL	325.47 ± 0.09 ^b^	248.28 ± 14.72 ^c^	573.75 ± 14.81 ^c^
	CM	267.55 ± 1.61 ^d^	141.42 ± 0.18 ^d^	408.97 ± 0.79 ^d^
*p*-coumaric acid	N	1043.82 ± 19.17 ^a^	134.85 ± 4.80 ^e^	1178.68 ± 14.37 ^b^
	C	1044.80 ± 25.03 ^a^	240.48 ± 14.49 ^a^	1285.28 ± 10.53 ^a^
	L	888.10 ± 1.12 ^d^	99.65 ± 0.14 ^f^	987.75 ± 0.98 ^e^
	CL	917.98 ± 1.30 ^c^	155.95 ± 0.05 ^d^	1073.93 ± 1.35 ^d^
	GL	921.16 ± 0.11 ^c^	201.07 ± 11.58 ^b^	1122.23 ± 11.70 ^c^
	CM	946.12 ± 2.27 ^b^	164.01 ± 0.24 ^c^	1110.12 ± 2.03 ^c^
Ferulic acid	N	267.82 ± 0.32 ^c^	23.01 ± 2.29 ^b^	290.83 ± 1.97 ^c^
	C	84.41 ± 0.08 ^f^	36.48 ± 12.41 ^a^	120.89 ± 12.34 ^f^
	L	179.22 ± 4.33 ^d^	15.65 ± 0.29 ^e^	194.86 ± 4.04 ^d^
	CL	125.52 ± 0.15 ^e^	18.41 ± 0.09 ^d^	143.93 ± 0.06 ^e^
	GL	317.03 ± 0.70 ^a^	26.64 ± 1.45 ^b^	343.67 ± 1.52 ^a^
	CM	303.86 ± 0.77 ^b^	21.69 ± 0.08 ^bc^	325.55 ± 0.63 ^b^
Sinapic acid	N	73.91 ± 5.31 ^d^	13.18 ± 1.18 ^b^	87.09 ± 4.13 ^c^
	C	104.76 ± 5.74 ^b^	17.40 ± 1.16 ^a^	122.15 ± 6.90 ^b^
	L	40.60 ± 0.37 ^f^	5.73 ± 0.01 ^d^	46.33 ± 0.38 ^e^
	CL	60.63 ± 0.27 ^e^	11.16 ± 0.18 ^bc^	71.80 ± 0.45 ^d^
	GL	211.39 ± 0.35 ^a^	12.08 ± 1.39 ^b^	223.46 ± 1.04 ^a^
	CM	84.80 ± 1.51 ^c^	5.65 ± 0.04 ^d^	90.45 ± 1.55 ^c^
In total	N	1807.89 ± 14.82 ^b^	532.60 ± 9.16 ^b^	2340.49 ± 5.66 ^b^
	C	1834.64 ± 31.21 ^b^	677.41 ± 21.57 ^a^	2512.05 ± 52.78 ^a^
	L	1569.57 ± 11.68 ^e^	175.83 ± 0.55 ^d^	1745.40 ± 11.13 ^d^
	CL	1611.14 ± 0.79 ^d^	542.87 ± 14.26 ^b^	2154.01 ± 13.47 ^c^
	GL	1991.55 ± 0.3 ^a^	547.60 ± 34.04 ^b^	2539.15 ± 34.34 ^a^
	CM	1729.24 ± 3.47 ^c^	371.38 ± 0.50 ^c^	2100.62 ± 2.96 ^c^

Treatments were as follows: N: NaCl; C: NaCl + CaCl_2_; L: NaCl + LaCl_3_; CL: NaCl + CaCl_2_ + LaCl_3_; GL: NaCl + GABA + LaCl_3_; CM: NaCl + CaCl_2_ + 3-mercaptopropionate (3-MP). Values bearing different letters in each form are significantly different (*p* < 0.05).

**Table 2:** Contents of individual and total isoflavonoids (µg/g DW) during germination (n = 3) in soybean sprouts.

	N	C	L	CL	GL	CM
Daidzin	430.8 ± 38.6 ^f^	634.3 ± 17.7 ^a^	510.3 ± 6.6 ^c^	601.9 ± 3.4 ^b^	499.8 ± 1.6 ^d^	452.4 ± 5.2 ^e^
Glycitin	26.7 ± 1.9 ^c^	33.9 ± 1.8 ^a^	27.3 ± 1.2 ^c^	31.8 ± 0.7 ^a^	28.5 ± 0.8 ^b^	25.5 ± 1.1 ^c^
Genistin	284.6 ± 4.7 ^d^	382.4 ± 10.6 ^a^	316.2 ± 0.7 ^c^	329.1 ± 1.0 ^b^	316.3 ± 1.6 ^c^	282.7 ± 6.1 ^d^
Malonyldaidzin	2620.3 ± 57.7 ^e^	5071.7 ± 72.6 ^a^	2456.8 ± 27.7 ^f^	3939.4 ± 49.4 ^b^	3461.7 ± 2.1 ^c^	3099.4 ± 3.1 ^d^
Malonylglycitin	268.0 ± 9.8 ^f^	356.8 ± 7.4 ^a^	329.4 ± 2.6 ^c^	337.7 ± 2.6 ^b^	297.6 ± 2.0 ^d^	279.5 ± 0.6 ^e^
Malonylgenistin	2670.8 ± 44.9 ^d^	2895.0 ± 26.3 ^b^	2513.6 ± 3.2 ^e^	2701.2 ± 16.4 ^c^	3021.1 ± 28.9 ^a^	2955.9 ± 44.1 ^b^
Daidzein	105.1 ± 6.6 ^b^	107.0 ± 0.7 ^b^	82.4 ± 1.0 ^c^	74.6 ± 0.1 ^d^	62.7 ± 13.4 ^e^	143.3 ± 12.6 ^a^
Genistein	40.9 ± 0.6 ^a^	37.1 ± 0.1 ^b^	ND *	ND	ND	42.5 ± 0.7 ^a^
Total	6447.0 ± 161.3 ^e^	9515.2 ± 61.3 ^a^	6236.6 ± 24.1 ^f^	8016.1 ± 37.4 ^b^	7687.9 ± 40.3 ^c^	7281.6 ± 45.8 ^d^

Treatments were as follows: N: NaCl; C: NaCl + CaCl_2_; L: NaCl + LaCl_3_; CL: NaCl + CaCl_2_ + LaCl_3_; GL: NaCl + GABA + LaCl_3_; CM: NaCl + CaCl_2_ + 3-mercaptopropionate (3-MP). Values bearing different letters in each line are significantly different (*p* < 0.05). * ND means not detected.

## Data Availability

Not applicable.
